# Helminth infections and practice of prevention and control measures among pregnant women attending antenatal care at Anbesame health center, Northwest Ethiopia

**DOI:** 10.1186/s13104-017-2609-6

**Published:** 2017-07-12

**Authors:** Melashu Balew Shiferaw, Amtatachew Moges Zegeye, Agmas Dessalegn Mengistu

**Affiliations:** 1 Amhara Public Health Institute, P.O. Box 641, Bahir Dar, Ethiopia; 2 North Shoa Zonal Health Department, Debre Birhan, Ethiopia; 3 Felege Hiwot Referral Hospital, Bahir Dar, Ethiopia

**Keywords:** Helminth, Prevention and control, Pregnant women, Northwest Ethiopia

## Abstract

**Background:**

Helminth infections have a terrible impact on child growth and development, and harm pregnant women. Regular treatment and long term preventive interventions are important measures to break the transmission routes. Hence, identifying the status of helminth infection and practices of prevention and control measures among pregnant women is important in different geographical areas of Ethiopia including our setting.

**Methods:**

A cross-sectional study was conducted on 180 pregnant women from March to June, 2015. About 2 g of stool was collected and examined to identify helminth infections. Proportions and risk factors of helminth infections were calculated using SPSS version 20.

**Results:**

Among the total 180 study participants, 38 (21.1% [95% CI 15.2–27.0%]) pregnant women had helminth infections. Hookworm and *Schistosoma mansoni* were the only identified helminth species. Thirty-six (20.0% [95% CI 14.3–25.7%]) and 4 (2.2% [95% CI 0.2–4.2%]) pregnant women had hookworm and *S. mansoni* infections, respectively. Of which, double infection (hookworm and *S. mansoni*) was found in two pregnant women. Only 32 (17.8%) pregnant women had proper hand wash practice after toilet, 48 (26.7%) drank treated water, and 40 (22.2%) wore shoes regularly. Those pregnant women who did not take albendazole or mebendazole dewormers (AOR 3.57; 95% CI 1.19–10.69; P 0.023) were more infected from helminth infections.

**Conclusions:**

This study showed that there was a high intestinal helminth infection among pregnant women, and low practice of prevention and control measures. Thus, prevention and control measures should be strengthened in the setting.

## Background

Helminthes are parasitic worms that cause morbidity and mortality in human and/or animal. Soil-transmitted nematodes namely roundworm (*Ascaris lumbricoides*), whipworm (*Trichuris trichiura*) and hookworms (*Necator americanus*/*Ancylostoma duodenale*), tapeworms such as *Taenia* spp. and *Hymenolepis* spp. and flukes are frequently found helminthes [1]. They are common among pregnant women in developing countries where poor water supply and poor environmental sanitation are available [2]. Hookworm alone infects approximately 44 million pregnant women in endemic areas [3]. Those pregnant women with anemia, commonly caused in low-income countries by hookworm, are three and a half times more likely to die during childbirth than women who are not anemic [4].

Helminthic infections in pregnancy impede child growth and development. The infection is often more severe and tends to result in a higher degree of parasitemia. Incidentally, the natural immune response to pregnancy causes women to be more susceptible to parasitic infections [4, 5].

As a prevention strategy, anthelminthic treatment can be dispensed through health services (maternal and child health and antenatal clinics), school health programs, and community interventions directed at other vulnerable groups (such as adolescent girls). Reinfection is common in this prevention strategy. Therefore, permanent control is the best option, which can be achieved through regular treatment accompanied by long-term key preventive interventions to break transmission routes through provision and use of a safe and adequate water supply, improvement of environmental sanitation, and good sanitation and hygiene habits [6].

Ethiopia is one of the high burden countries of the helminth infections. In southern Ethiopia, previous studies documented that higher prevalence of helminthic infections among pregnant women [7, 8]. However, the distribution of helminth infection varies in different geographical areas according to studies conducted in primary school-age children and health care attendants in Northwest Ethiopia [9, 10]. Hence, identifying the status of helminth infection and practices of prevention and control measures among pregnant women is important in different settings of Ethiopia including our setting.

## Methods

### Study area and study subjects

This cross-sectional study design was conducted to determine the burden of helminth infections, and identify practices of prevention and control measures among pregnant women attending the antenatal care in Anbesame health center, South Gondar, Ethiopia. The study was conducted from March to June, 2015. In this area, the climate is woina dega, and has 2077 meters above sea level. The mean annual rain fall and temperature of the area was 1300 mm^3^ and 26 °C, respectively [11]. The Anbesame health center antenatal clinic provides reproductive health related follow up services to pregnant women. A total of 752 pregnant women got antenatal care (ANC) service in 2015 alone. All pregnant women attending antenatal care in the health center were study populations. The study subjects were those who had got ANC service during the study period. Those pregnant women who took deworming within 1 month were excluded by considering the use of deworming in this period may result more negative findings that could further under estimate the true prevalence of helminth infections.

### Sample size and sampling technique

Sample size was determined using single population proportion formula by considering the following assumptions: 9.6% prevalence of helminth infection among pregnant women from Azezo health center, North Gondar, Ethiopia [12], 0.05 significant level (α), 95% level of confidence, 5% margin of error and 10% non-response rate. Using the formula: n = (1.96)^2^ * P * (1 − P)/(0.05)^2^; n = (1.96)^2^ * 0.096 * (1 − 0.096)/(0.05)^2^ = 134, and adding 10% non-response rate, the minimum sample size was 148. However, the pregnant women visiting the clinic within the study period were 180. Therefore, all the 180 pregnant women were included to increase representativeness of the population. Those women were selected consecutively based on the arrival in the ANC clinic of the health center.

### Data collection and laboratory diagnosis

Socio-demographic data and practices of prevention and control measures of study participants were collected using a standardized questionnaire. About 2 g of stool specimen was collected using a clean plastic stool container. To identify motile intestinal parasites, a portion of stool sample was examined using direct wet mount with saline within 15 min after the sample collection. The remaining stool specimen was processed using formol-ether concentration technique and examined to identify ova of parasites [13]. Laboratory technologists that had previous experience on stool examination examined the testing. The specimens were read by at least two laboratory technologists for the true quality testing.

### Quality assurance

Quality of the data was ensured by implementing the following activities. Two days training was given for data collectors on how to interview study participants; stool specimen was properly labeled, and standard operating procedure was followed during specimen collection, processing and examination; and completeness of the data was checked daily.

### Statistical analysis

Data were entered, cleaned and analyzed using SPSS version 20. Proportions of intestinal helminth infections and practices of prevention and control measures were computed. Results were presented in figure, tables and texts. Variables having P value ≤0.2 in the bivariate analysis were further entered into the multivariate analysis to control the effects of confounders. Associations between helminth infections and data of socio-demographic and practices of prevention and control measures were determined using multiple logistic regressions. P value <0.05 was the cut point for significant association.

## Results

### Characteristics of study participants

Of the total 180 participants, 108 (60.0%) pregnant women were between 20 and 30 years of age group with a mean (±SD) of 26.39 (±5.88) years. Almost all of, 176 (97.8%), the study participants were married. Participants with illiterate and elementary educational status were 132 (73.3%) and 36 (20.0%), respectively. About 89% of the participants were house wives (Table 1).

**Table 1:** Characteristics of study participants, Anbesame health center, 2015

Characteristics	Response	Frequency	Percent
Age in years	<20	40	22.2
	20–30	108	60.0
	>30	32	17.8
Marital status	Married	176	97.8
	Single	2	1.1
	Divorced	2	1.1
Educational status	College and above	6	3.3
	High school	6	3.3
	Elementary	36	20.0
	Illiterate	132	73.3
Occupation	Farmer	8	4.4
	Government employee	8	4.4
	Merchant	4	2.2
	House wife	160	88.9
Family size	2	40	22.2
	3	18	10.0
	4	20	11.1
	5	22	12.2
	6	28	15.6
	7	34	18.9
	8	14	7.8
	9	4	2.2

### Practice of prevention and control measures

Only 48 (26.7%) pregnant women drank chlorine treated water from the pipe source. Most of the pregnant women, 162 (90.0%), had experience of open field defecation. Hand washing after toilet was practiced by 32 (17.8%) pregnant women. Less than half, 40 (22.2%), of the participants wore shoes regularly. One hundred thirty-four (74.4%) pregnant women did not take albendazole (400 mg) or mebendazole (500 mg) dewormers (Table 2).

**Table 2:** Practice of prevention and control measures, Anbesame health center, 2015

Variables	Response	Number	%
Drank chlorine treated water	No	132	73.3
	Yes	48	26.7
Had latrine	No	84	46.7
	Yes	96	53.3
Hand wash after toilet	No	148	82.2
	Yes	32	17.8
Walking barefoot	No	40	22.2
	Yes	140	77.8
Took deworming from the last 1 month to the last 1 year	No	134	74.4
	Yes	46	25.6
Iron supplementation	No	110	61.1
	Yes	70	38.9
Open field defecation	No	18	10.0
	Yes	162	90.0
Water contact	No	30	16.7
	Yes	150	83.3

### Helminth infections

Among the participants, 38 (21.1% [95% CI 15.2–27.0%]) pregnant women had helminth infections. Hookworm and/or *Schistosoma mansoni* were the identified helminth’s species in this study. Thirty-six (20.0% [95% CI 14.3–25.7%]) pregnant women had hookworm infections, and 4 (2.2% [95% CI 0.2–4.2%]) pregnant women had *S. mansoni* infections. Of which, co-infection (hookworm and *S. mansoni*) was found in two pregnant women. All participants with *S. mansoni* infection had water contact history. Those who did not take albendazole (400 mg) or mebendazole (500 mg) dewormers (AOR 3.57; 95% CI 1.19–10.69; P 0.023) had significantly higher helminth infections (Table 3).

**Table 3:** Associated factors of helminth infection, Anbesame health center, 2015

Variables	Categories	Helminth infection		Crude OR (95% CI)	Adjusted OR (95% CI)	P value
		No	Yes			
Age (years)	<20	30	10	1.0 (0.34–2.93)	–	–
	20–30	88	20	0.68 (0.27–1.74)	–	–
	>30	24	8	1	–	–
Drank treated water	No	102	30	1.47 (0.62–3.48)	–	–
	Yes	40	8	1	–	–
Hand washing after toilet	No	116	32	1.20 (0.45–3.15)	–	–
	Yes	26	6	1	–	–
Took deworming from the last 1 month to the last 1 year	No	100	34	3.57 (1.19–10.69)	3.57 (1.19–10.69)	0.023
	Yes	42	4		1	
Walking barefoot	No	30	10	1.33 (0.58–3.05)	–	–
	Yes	112	28	1	–	–

The blank cells in the table are outputs of backward LR method of the multiple logistic regressions

*COR* crude odds ratio, *AOR* adjusted odds ratio, *CI* confidence interval

## Discussion

In this study, the overall prevalence of helminth infection was 21.1%. Of which, hookworm infection accounted for almost all of (20%) the helminth infections among pregnant women in our setting. Hookworm infections cause mechanical laceration and enzymatic damage to the mucosa of the small intestine leaving behind small bleeding lesions [14]. The gastrointestinal blood loss, malabsorption and appetite inhibition may further aggravate the iron, zinc and protein energy deficiencies during pregnancy [15–17]. Pregnant women with hookworm infection are at high risk of giving birth to low birth-weight babies, of poor milk production, and of birthing babies who fail to thrive, and are three and a half times more likely to die during childbirth than women who are not anemic [4, 16]. In addition, helminthic infections may serve as important factors in the transmission of human immunodeficiency virus [18].

The hookworm prevalence found from this study is lower compared to the hookworm infections reported in Gilgel (29.4%) [8] and in Nigeria (35.8%) [19]. Moreover, previous studies in southern Ethiopia [7, 8] reported high prevalence of Ascaris and Trichuris as well as other helminth species. Surprisingly, this study detected only two species (Hookworm and/or *S. mansoni*). This may be due to the difference in distribution of helminth species in different geographical areas in addition to method difference that might underestimate the detection of helminth infection.

However, this finding is higher compared to studies that reported hookworm infection of 4.7% in Northwest Ethiopia [12], 7.0% in Southern Ethiopia [7], 3.9% in Kenya [20] and 8.1% in Venezuela [21]. The high rates of hookworm infection are mostly indicative of faecal pollution of soil and domestic water supply around homes due to poor sanitation and improper sewage disposal [2]. Other practices such as hand washing, disposal of refuse, personal hygiene, and wearing of shoes, when not done properly, may contribute to the infection or picking of infective forms of the worms from the environment [22]. Our study has also found a low practice of prevention and control measures that only 17.8% had hand washing practice after toilet, 26.7% drank treated water, and 22.2% wore shoes regularly.

In areas where helminths are common, deworming activities can be done once or twice per year to the population at risk including deworming for pregnant women after the first trimester of pregnancy to reduce severe maternal anemia, increase birth weight and reduce infant mortality. Both albendazole (400 mg) and mebendazole (500 mg) offer the further advantage that they can be administered as a single dose to all individuals over 2 years of age. They are also safe for children between 1 and 2 years of age (although the dose of albendazole has to be reduced to 200 mg) [6]. However, we found a very low coverage of deworming that 74.4% of the pregnant women did not take albendazole or mebendazole deworming. The low deworming coverage caused significantly higher helminth infection in pregnant women in our setting (AOR 3.57; 95% CI 1.19–10.69; P 0.023).

As limitation, due to budget shortage, this study was done based on faecal samples examination by wet mount (direct smear) and concentration method only without consideration of other alternative techniques such as Kato-Katz, Harada Mori and Baermann techniques, which are important to detect intestinal parasite species. This may underestimate the sensitivity of identifying helminth infections. The sample size calculation was only to estimate the prevalence and probably the sample size was not adequate to identify risk factors except for history of deworming.

## Conclusions

This study showed that there was high helminth infection, and low practice of prevention and control measures among pregnant women. Thus, intestinal helminth prevention and control measures should be strengthened in the setting.

## Abbreviations

ANC: antenatal care
AOR: adjusted odds ratio
CI: confidence interval
COR: crude odds ratio
